# Individuals with cerebral palsy show altered responses to visual perturbations during walking

**DOI:** 10.3389/fnhum.2022.977032

**Published:** 2022-09-08

**Authors:** Ashwini Sansare, Maelyn Arcodia, Samuel C. K. Lee, John Jeka, Hendrik Reimann

**Affiliations:** ^1^Department of Physical Therapy, University of Delaware, Newark, DE, United States; ^2^Department of Kinesiology and Applied Physiology, University of Delaware, Newark, DE, United States

**Keywords:** sensorimotor integration, locomotor control, balance, virtual reality, visual reliance

## Abstract

Individuals with cerebral palsy (CP) have deficits in processing of somatosensory and proprioceptive information. To compensate for these deficits, they tend to rely on vision over proprioception in single plane upper and lower limb movements and in standing. It is not known whether this also applies to walking, an activity where the threat to balance is higher. Through this study, we used visual perturbations to understand how individuals with and without CP integrate visual input for walking balance control. Additionally, we probed the balance mechanisms driving the responses to the visual perturbations. More specifically, we investigated differences in the use of ankle roll response i.e., the use of ankle inversion, and the foot placement response, i.e., stepping in the direction of perceived fall. Thirty-four participants (17 CP, 17 age-and sex-matched typically developing controls or TD) were recruited. Participants walked on a self-paced treadmill in a virtual reality environment. Intermittently, the virtual scene was rotated in the frontal plane to induce the sensation of a sideways fall. Our results showed that compared to their TD peers, the overall body sway in response to the visual perturbations was magnified and delayed in CP group, implying that they were more affected by changes in visual cues and relied more so on visual information for walking balance control. Also, the CP group showed a lack of ankle response, through a significantly reduced ankle inversion on the affected side compared to the TD group. The CP group showed a higher foot placement response compared to the TD group immediately following the visual perturbations. Thus, individuals with CP showed a dominant proximal foot placement strategy and diminished ankle roll response, suggestive of a reliance on proximal over distal control of walking balance in individuals with CP.

## Introduction

Individuals with cerebral palsy (CP) are at a high risk for falls, with about 35% reporting daily falls and 30% reporting weekly or monthly falls (Boyer and Patterson, 2018). These falls not only lead to serious injuries to the head, spine, and limbs, but also affect psycho-social health, resulting in fear, embarrassment, and the feeling of powerlessness in individuals with CP (Morgan et al., 2015). To maintain upright balance, the nervous system uses sensory information to determine how the body moves through space and generates appropriate motor command to modulate that movement and keep the body upright. Thus, both sensory and motor deficits can impair the functioning of this sensorimotor control loop and contribute to balance problems and fall risk. Rehabilitation approaches generally focus on the motor aspect of balance control, and most studies in CP examine the postural control strategies due to altered muscle recruitment strategies (Roncesvalles et al., 2002) and kinetics (Chen and Woollacott, 2007), often using mechanical perturbations (Tracy et al., 2019; Crenshaw et al., 2020). However, the effect of sensory deficits on sensorimotor control of balance in walking is currently not well understood.

Individuals with CP have deficits in processing of somatosensory and proprioceptive information. CP is associated with altered sensorimotor cortical activation and disrupted sensory white matter connections (Hoon et al., 2009; Kurz et al., 2014, 2015) and poor performance on clinical sensory tests, such as reduced scores in two-point discrimination, light touch, and hip and ankle joint position sense (Damiano et al., 2013; Zarkou et al., 2020). There is increasing evidence that these sensory deficits are linked to deficits in balance control and walking performance in CP. Mobility impairments in CP, including slower walking speed and shorter steps, are associated with abnormal somatosensory cortical activity (Kurz et al., 2015). Additionally, poor performance on clinical sensory tests at the legs, such as impaired two-point discrimination, light touch, hip and ankle joint position sense, have moderate to strong relationships with reduced balance parameters such as increased postural sway, poor scores in the Balance Evaluation Systems Test (BESTest), as well as with reduced walking performance such as slower gait speed, shorter step length, shorter 6 min Walk test distance (Damiano et al., 2013; Zarkou et al., 2020). These findings suggest a causal link of deficits in somatosensory and proprioceptive processing and to impairments in postural control in CP.

Individuals with CP use visual information to compensate for deficits in somatosensory and proprioceptive processing. Occluded vision results in higher errors during single plane upper and lower extremity movements in individuals with CP (Wingert et al., 2009). Individuals with CP exhibit larger and more variable body sway in maintaining upright stance in response to visual manipulation of the surrounding environment than typically developing peers (Barela et al., 2011; Slaboda et al., 2013). Removal of visual input worsens the crouch stance by increasing hip-knee flexion in individuals with CP, suggesting that visual input is important for maintaining standing posture in individuals with CP (Lidbeck et al., 2016). The degree of visual dependency during standing is associated with abnormal balance strategies in CP (Yu et al., 2020) and an increased risk of impaired development in posture, spatial awareness, and movement skills (Sonksen and Dale, 2002). Visual dependency is associated with poor balance and increased fall risk in older adults and people with vestibular disorders (Lord and Webster, 1990; Suárez et al., 2001; Lee, 2017), suggesting that vision can only compensate for deficits in other sensory modalities to a certain degree.

While increased reliance on visual information over proprioception in individuals with CP was established in single plane upper and lower extremity movements (Wingert et al., 2009) and during standing (Slaboda et al., 2013), the implications for balance control during walking have so far not been studied. Though it is reasonable to assume that sensory integration is similar between standing and walking, the effect of visual dependency on balance in walking not been studied in individuals with CP. The biomechanics of the walking human body are substantially more complex than in standing. The body configuration changes substantially during the gait cycle, requiring different biomechanical mechanisms to modulate body movement and maintain upright balance (Reimann et al., 2018b). Due to this biomechanical difference, it is unclear if results from studies on sensory processing for balance in standing will translate to walking. Moreover, the majority of falls in individuals with CP occur in walking and other dynamic activities such as turning, bending, and lifting (Morgan et al., 2015), rather than in standing. Hence it is critical to investigate how the visual dependency seen in standing balance affects balance control during walking in individuals with CP.

A typical response to a visual fall stimulus is to move the center of mass (CoM) away from the direction of the fall. People react to a leftward fall stimulus by moving their body to the right. Visual perturbations during walking in older adults resulted in increased CoM motions, implying that aging induced degradations in proprioception may lead to reliance on visual feedback (Franz et al., 2015). Our hypothesis that individuals with CP compensate for proprioceptive deficits by relying more on visual information predicts that responses to visual fall stimuli during walking will be larger in individuals with CP, because they rely more on visual feedback than on the proprioceptive or vestibular information indicating that the body is not actually falling.

The CoM response to visual fall stimuli is generated by two different biomechanical mechanisms in healthy adults. Immediately following a fall stimulus, they change the foot placement location of the next step in the direction of the perceived fall, which changes the lever arm of the gravitational force and accelerates the body in the opposite direction (Reimann et al., 2018b). This is known as the “foot placement mechanism” for balance control (Wang and Srinivasan, 2014; van Dieën and Bruijn, 2018). The second mechanism is to use lateral ankle musculature during single stance to actively pull the body to the side (Hof and Duysens, 2018; Reimann et al., 2018a), referred to as “ankle roll mechanism.” The ankle roll mechanism is important for balance control in walking. Although it is limited in extent by the contact area under the stance foot and usually small, it can often be applied earlier than the foot placement mechanism, which is limited by the step time. Modeling results (Reimann et al., 2017) indicate that in the absence of ankle response, a larger foot placement response is needed to upright balance. In standing balance control, individuals with CP tend to favor responses with the hip joints over ankle joint responses, activating the proximal musculature around the hips rather than showing the distal-to-proximal strategy usually observed in neurotypical adults (Burtner et al., 1999; Chen and Woollacott, 2007). We hypothesize that individuals with CP show a similar preference of proximal over distal responses in walking balance control, predicting that they will show reduced response in the ankle roll mechanism and increased response in the foot placement mechanism.

In this study, we investigated how individuals with CP integrate visual information for walking balance control by inducing visual fall stimuli in a virtual reality environment in individuals with and without CP. We measure the overall CoM response to the fall stimuli, and the responses in the foot placement and ankle roll balance mechanisms that generate the CoM movement. We hypothesize that people with CP compensate for proprioceptive and somatosensory deficits by relying more on visual information for balance and that they will favor proximal over distal mechanisms to control body movement. These hypotheses predict that individuals with CP (i) will display a larger CoM response to visual perturbations and (ii) will show larger foot placement response and reduced ankle roll response than age- and sex-matched peers.

## Materials and methods

### Participants

Seventeen ambulatory individuals and adolescents with spastic diplegic or hemiplegic CP were recruited through the CP clinic at local hospitals. We specifically recruited individuals with Gross Motor Function Classification System (GMFCS) levels I–II (Palisano et al., 1997) so that they would be able to complete our visual perturbation paradigm without relying on a handrail for tactile or visual cues. Seventeen typically developing (TD) individuals, who were age-matched (±6 months) and sex-matched with the CP group, were recruited through flyers, local advertisements, and social media. The protocol was approved by the University of Delaware Institutional Review Board, and informed parental consent and assent were obtained. All participants were screened by a physical therapist for the inclusion and exclusion criteria (Table 1). Of note, we only included individuals with normal or corrected to normal vision (such as with glasses or contact lenses) and excluded any individuals with any ocular impairments as well as known diagnosis of cerebral visual impairment (CVI). Additionally, all of our patients had at least 5 degrees of active motion in plantarflexion and dorsiflexion, at least 10 degrees of active knee flexion and at least 10 degrees of active hip flexion and extension. To analyze effects of laterality, we determined the *more affected side* as the one with hemiplegia in individuals with hemiplegic CP, the one self-identified as the more affected side in individuals with diplegic CP, and the non-dominant side for individuals with TD. The dominant side in the TD group was self-determined by the participants as their preferred lower limb of use during daily activities.

**TABLE 1:** Inclusion and exclusion criteria.

Inclusion	Exclusion
• Age 8–24 years • Diagnosis of spastic diplegic or hemiplegic CP • GMFCS classification level I or II (ability to walk independently with using any assistive device) • Visual, perceptual, and cognitive/communication skills to follow multiple step commands • Seizure-free or well controlled seizures • Ability to communicate pain or discomfort during testing procedures • Parental/guardian consent and child assent/consent	• Diagnosis of athetoid, ataxic, or quadriplegic CP • Significant scoliosis (scoliometer angle > 9°) • History of selective dorsal root rhizotomy • Botox injections in the lower limb within the past 6 months • Severe spasticity of the lower extremity muscles (e.g., a score of 4 on the Modified Ashworth Scale) • Severely limited range of motion/irreversible muscle contractures • Lower extremity surgery or fractures in the year prior testing* • Joint instability or dislocation in the lower extremities* • Marked visual or hearing deficits*

*Denotes criteria applicable to TD group.

### Instrumentation

**FIGURE 1:**
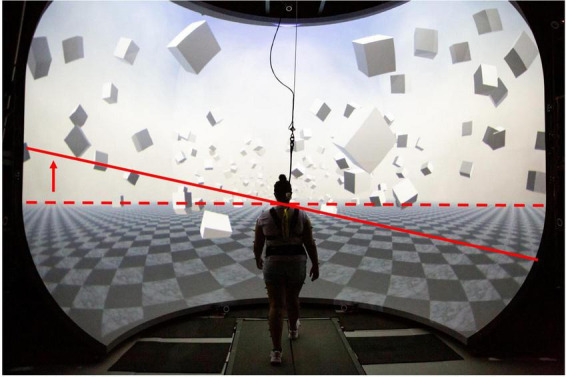
Virtual Reality Environment: The solid red line shows the tilt of the horizon (dashed) during the visual fall perturbation for a left sided virtual fall. Similar degree of tilt on the opposite side was generated for a right sided fall.

All participants walked on a split-belt treadmill (Bertec Inc., Columbus, Ohio, United States) within a virtual reality environment projected on a domed screen covering the participant’s complete field of vision. The virtual scene consisted of floating cubes that formed a 4-m wide, infinitely long corridor along a checkered floor (Unity3d, Unity Technologies, San Francisco, CA, United States). The treadmill was self-paced using a custom Labview program (National Instruments Inc., Austin, TX, United States), with the speed of the treadmill adapting in real time to the participant’s self-selected speed by keeping the midpoint between the posterior superior iliac spine markers in the anterior-posterior center of the treadmill. Additionally, the perspective in the virtual world was linked to the markers on the forehead and adapted in real time to the participant’s head movement. Figure 1 shows the virtual reality cave and the virtual scene used in this experimental protocol. These features created a compelling and immersive virtual reality experience similar to what one would experience when walking over-ground. Participants were in a safety harness at all times that protected them against falls but did not provide support during normal walking.

To induce visual perturbations, the virtual scene was rotated around the central anterior-posterior axis of the treadmill with an angular acceleration of 45°/s^2^ for 600 ms, starting at heel-strike of either foot, then remained tilted for 2,000 ms and was reset to the horizontal axis over the next 1,000 ms with constant angular velocity. This rotation generates optical flow on the participant’s retina that is similar to the optical flow of falling sideways around the stance foot contact point. These perturbations were triggered at pseudo-randomly selected heel strikes of either foot. Each such trigger was followed by a 10–12 step washout period between the reset of the visual scene and the next trigger. To determine the effect of the visual stimulus relative to unperturbed walking, we alternated between periods of actual and sham stimulation. The average walking pattern of the sham stimulation periods was used as control for each participant. A sham stimulation was a period of 10–12 steps where the participant would not receive any visual perturbation. i.e., they continued walking in an unperturbed manner for these steps. We have used this visual stimulation paradigm extensively in our previous work (Reimann et al., 2018a,b; Fettrow et al., 2019b).

### Protocol

Each participant was given at least two 2-min practice trials to get accustomed to the self-paced treadmill and the virtual environment, one trial without visual perturbations, and another practice trial with visual perturbations. Before beginning the practice walking trial with visual perturbations, the participants were given a demonstration of the visual perturbations to each side while in standing. We asked participants if they felt comfortable with the system after these two practice trials and offered additional practice on request.

After completing the practice phase, participants performed ten 2-min walking trials, with a 15 s ramp-up period prior to the start of the 2 min to reach steady-state walking. Participants received visual fall stimuli as described above during the 2-min period. After each trial, the treadmill was stopped, and participants were offered a rest break.

### Data analysis

Full body kinematics were measured using a 13-camera motion capture system (Qualysis Inc., Gothenberg, Sweden) and a full body Plug-in Gait marker set (Davis et al., 1991) with six additional markers on anterior thigh, anterior tibia, and 5th metatarsal head bilaterally. For the static calibration pose at the beginning of the collection, an additional six markers were placed on bilateral medial femoral epicondyles, medial malleoli, and the tip of the first distal phalanx of the foot. Heel strikes were tracked using the position of marker on the heel of each foot and were defined as the maxima of the anterior-posterior position of the heel-marker. We recorded electromyography (EMG) signals from peroneus longus, using Cometa’s PicoEMG sensors (Bareggio, Italy) with hydrogel 30 × 24 mm Covidien Kendall electrodes with SENIAM guidelines for placement. To normalize EMG, we divided by the average signal for each muscle across all the control steps. Marker data were recorded at 200 Hz, EMG at 2,000 Hz and ground reaction forces and moments at 1,000 Hz. Force plate data was low pass filtered with a 4th order Butterworth filter at a cut-off frequency of 20 Hz. EMG data was rectified, then low pass filtered with a 4th order Butterworth filter at a cut-off frequency of 6 Hz. Inverse kinematics for a 15-segment biomechanical model were computed using OpenSim 4.0 (Lu and O’Connor, 1999). The data was further processed using custom scripts in MATLAB (The MathWorks, Inc., Natick, Massachusetts, United States).

We analyzed the first eight steps following a visual stimulus. All data was time-normalized to 100 time points per step. For visual presentation in the figures, we scaled the normalized time to the average step time for each step. For all trajectories, the mean of control data for the same stance foot was subtracted from the stimulus data to estimate the response due to the visual stimulus. For spatial variables, the data from stimuli triggered by a left heel strike were inverted for body symmetry, such that positive values refer to the direction *toward the leg that triggered the stimulus* and negative refers to the direction *away from the leg that triggered the stimulus*. We determined heel strike and push off events as the maxima and minima of the heel marker relative to the midpoint of the pelvis markers in anterior-posterior direction.

### Outcome measures

The primary outcome measure to quantify the overall response to visual perturbations was the area under the curve (AUC) of the medio-lateral CoM excursion. We defined the CoM excursion as the difference between the average CoM for the perturbed steps from control for each participant and integrate over the eight steps following the heel strike that triggered a stimulus. We also determined the peak of the CoM excursion over the same period and used the magnitude of the peak to quantify the extent and the delay between the peak and the triggering heel strike to quantify the timing of the response.

To quantify the ankle roll, we calculated the subtalar angle and the peroneus longus EMG at the stance leg, integrated over the first single-stance period following perturbation onset.

To quantify the foot placement response, we first accounted for the normal variations of the foot placement based on the kinematics of the CoM. We fitted a linear model with the position and velocity of the CoM relative to the stance foot ankle at midstance as predictor and the medial-lateral foot placement as outcome to the data from all control steps for each participant (Wang and Srinivasan, 2014). To estimate the change in foot placement induced by the visual stimulus, we used this linear model to predict the foot placement location from the preceding CoM state, and then subtracted this prediction from the observed foot placement location (Reimann et al., 2017; Arvin et al., 2018). In addition to the foot placement change at the first post-perturbation step, we also calculated the average foot placement response over the first three post-perturbation steps as a measure of the overall foot placement response, because we expected a delayed response in individuals with CP. These outcome measures have been previously used to assess balance response to visual perturbations (Vlutters et al., 2016; Reimann et al., 2018b; Fettrow et al., 2019b).

In addition to these primary outcome measures, we also calculated body-mass index (BMI), and the average cadence, step length, step width, step time, and velocity. Step time is the time between consecutive heel strikes, cadence the inverse of the step time, step length and width the difference between the locations of the ankle markers at consecutive heel-strikes and velocity is step length divided by step time.

### Statistical analysis

Data were analyzed using separate two-way mixed ANOVAs, with group (CP, TD) as the between subject factor and side (more affected, less affected) as the within-subject factor. We expected a group by side interaction where participants with CP would perform worse on the affected side when compared to their TD counterparts. We assessed assumptions of homoscedasticity and normality, respectively, by Levene’s and Shapiro-Wilk tests in addition to visual examination. Between group differences for baseline characteristics such as age, body mass index (BMI), cadence, velocity, step length, step time, and step width were assessed using paired samples *t-*test.

## Results

All participants responded to the visual fall stimuli without having to use the safety harness or stepping off the treadmill.

Most participants (31 out of 34) responded to the visual perturbations by moving their CoM away from the direction of virtual fall, as expected. Two participants from the CP group responded to the fall stimulus by moving their CoM in the opposite direction, toward the perceived fall rather than away from it. One participant from the CP group responded by lunging forward and crouching instead of moving in the mediolateral direction. We chose to exclude these three participants and their respective controls from the statistical analysis, because the CoM response of these three participants was atypical and not representative of the remaining participants in their cohort, so that averaging across the whole group would distort the results and not be representative of the group-wide behavior. Data from these individuals is included in Supplementary Figure 1. Additionally, EMG data from two participants from the TD group and one participant from the CP group were excluded from the analysis due to technical issues during the data collection. The final number of participants analyzed was 14 CP and 14 TD, with the exception of the peroneal EMG analysis, which included data from 13 CP and 12 TD.

The demographic and spatiotemporal gait parameters for both groups are reported in Table 2. The BMI of the CP group was significantly lower than that of the TD group. Additionally, their cadence was significantly higher, and step length and step time significantly lower than that of the TD group. No significant between-group differences were found for age, velocity and step width. Out of the fourteen individuals in the CP group, there were four participants with hemiplegia and the remaining 10 had diplegia.

**TABLE 2:** Mean, standard deviation (SD), and *p*-values for the difference between the CP ad TD groups for demographic and spatiotemporal gait variables.

	CP (*n* = 14)	TD (*n* = 14)	
	Mean ± SD	Mean ± SD	*p*-value
Age	16.3 ± 4.3	16.1 ± 4.2	0.27
BMI	19.0 ± 3.1	23.6 ± 4.7	0.011
Cadence (steps/min)	115 ± 10	106 ± 9	0.007
Velocity (m/s)	0.966 ± 0.186	1.026 ± 0.166	0.277
Step width (m)	0.159 ± 0.065	0.124 ± 0.034	0.091
Step length: More affected side (m)	0.502 ± 0.087	0.577 ± 0.075	0.012
Step length: Less affected side (m)	0.501 ± 0.087	0.579 ± 0.072	0.008
Step time: More affected side (s)	0.528 ± 0.047	0.572 ± 0.051	0.01
Step time: Less affected side (s)	0.528 ± 0.047	0.570 ± 0.050	0.007

### Center of mass response

**FIGURE 2:**
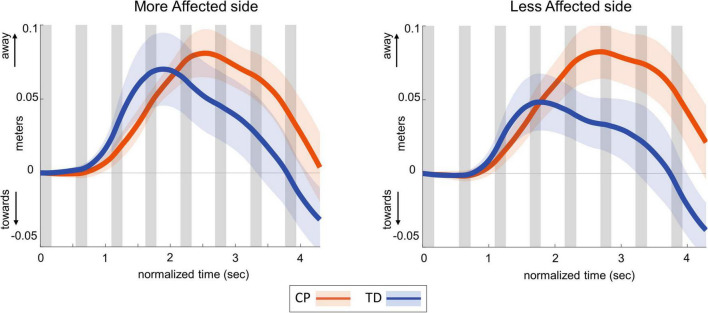
Group average trajectories for medio-lateral center of mass excursion in response to visual fall stimuli in CP (orange) and TD (blue). Thick gray line along zero at *X*-axis indicates the mean of control (no fall stimulus) steps, which is subtracted from stimulus data. Shaded areas around each trajectory represent 95% confidence interval. *X*-axis shows 8 steps, time-normalized to 100 timepoints per steps, with double-stance (gray shading) and single-stance (no shading).

**FIGURE 3:**
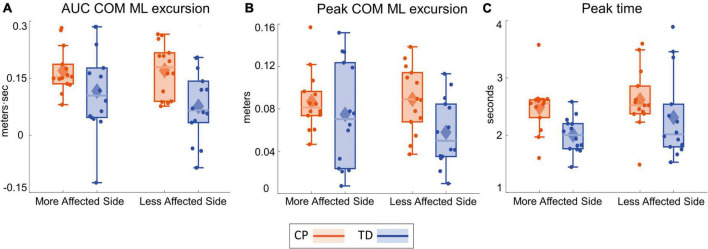
Box and whisker plots, with scattered dots indicating each subject, for AUC ΔCOM M-L excursion **(A)**, Peak ΔCOM M-L excursion **(B)** and Peak time of ΔCOM M-L excursion **(C)** for CP (orange) and TD (blue) groups on the more affected (left) and less affected (right) side.

Table 3 presents the descriptive statistics for all outcome variables, separated by factors group and side. Figure 2 shows the average ML CoM excursion over eight post-perturbation steps. Visual perturbations resulted in an overall larger CoM excursion in individuals with CP. Figure 3 shows box and whisker plots for AUC CoM excursion, peak CoM excursion, and peak time. There was a significant main effect for group for AUC ML CoM excursion (*p* = 0.017) with the CP group showing a higher CoM excursion than the TD group (Figure 3A), indicating an overall magnified response in the CP group to visual perturbations. The CP group reached higher average peak CoM excursion than TD on both sides (Figure 3B), but this group effect was not statistically significant (*p* = 0.093), nor was the side or group by side interaction. There was a significant group effect for the peak timing of CoM excursion (*p* = 0.031), with the CP group averaging higher peak times compared to the TD group, i.e., they reached peak CoM excursion later than TD (Figure 3C). There was also a significant main effect for side for peak timing (*p* = 0.042), where both CP and TD groups reached the peak CoM excursion quicker on their more affected side compared to their less affected side. Full details of the statistical analysis are provided in Supplementary Table 1.

**TABLE 3:** Mean and 95% confidence interval for CP and TD groups on the more affected and less affected side.

	Group	More affected side			Less affected side		
		Mean	95%CI		Mean	95%CI	
AUC ML CoM excursion (m⋅s)	CP	0.170	0.136	0.203	0.171	0.131	0.211
	TD	0.116	0.052	0.181	0.076	0.024	0.128
Peak ML CoM excursion (m)	CP	0.087	0.071	0.103	0.089	0.071	0.107
	TD	0.075	0.046	0.104	0.058	0.039	0.076
Peak time (s)	CP	2.484	2.229	2.738	2.615	2.309	2.922
	TD	2.005	1.829	2.182	2.310	1.883	2.737
Step placement-1st step (m)	CP	–0.002	–0.004	0.000	–0.003	–0.005	–0.001
	TD	–0.006	–0.009	–0.004	–0.004	–0.007	0.000
Step placement-averaged over 3 steps (m)	CP	–0.008	–0.011	–0.005	–0.007	–0.010	–0.004
	TD	–0.002	–0.005	0.001	–0.002	–0.005	0.002
Subtalar angle (degrees)	CP	–0.031	–0.100	0.038	0.065	0.002	0.128
	TD	0.178	0.032	0.325	0.057	–0.080	0.195
Peroneal EMG (%)	CP	–0.017	–0.044	0.009	–0.002	–0.016	0.013
	TD	–0.012	–0.024	–0.001	–0.011	–0.042	0.019

### Ankle roll response

**FIGURE 4:**
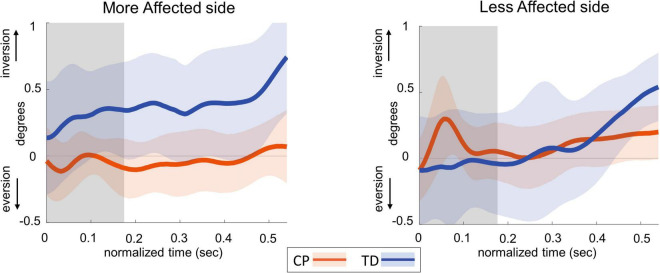
Group average trajectories for subtalar angle during the first post-stimulus step following a visual fall perturbation in CP (orange) and TD (blue). Thick gray line along zero at *X*-axis indicates the mean of control (no fall stimulus) steps, which is subtracted from stimulus data. Shaded areas around each trajectory represent 95% confidence interval. *X*-axis shows 8 steps, time-normalized to 100 timepoints per steps, with double-stance (gray shading) and single-stance (no-shading). Positive and negative *Y*-axis indicates inversion and eversion, respectively.

**FIGURE 5:**
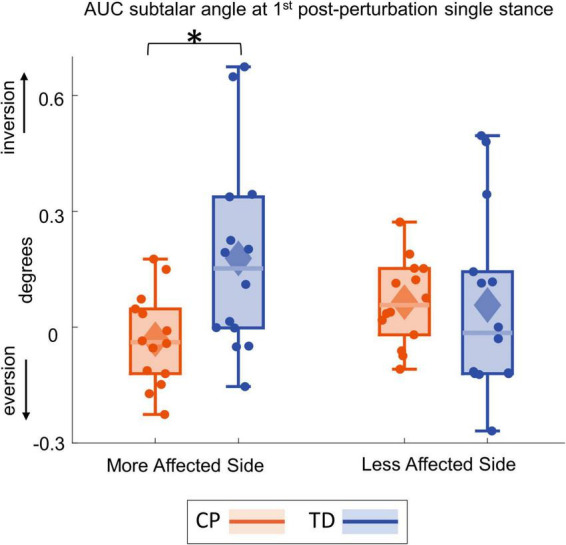
Box and whisker plots, with scattered dots indicating each subject, for AUC Δsubtalar angle for CP (orange) and TD (blue) groups on the more affected (left) and less affected (right) side. Asterisk indicates *p* < 0.05 for *post hoc* pairwise comparisons.

**FIGURE 6:**
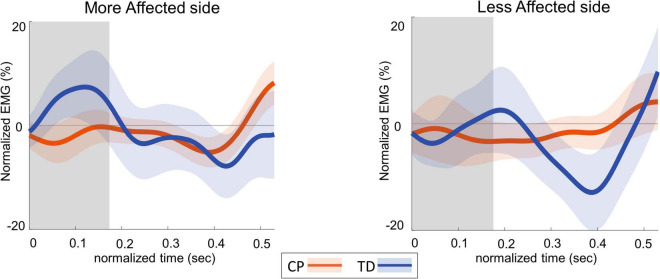
Group average trajectories for peroneal EMG during the first post-stimulus step following a visual fall perturbation in CP (orange) and TD (blue). Thick gray line along zero at *X*-axis indicates the mean of control (no fall stimulus) steps, which is subtracted from stimulus data. Shaded areas around each trajectory represent 95% confidence interval. *X*-axis shows 8 steps, time-normalized to 100 timepoints per steps, with double-stance (gray shading) and single-stance (no shading). *Y*-axis indicates EMG data normalized to average EMG of no perturbation steps.

**FIGURE 7:**
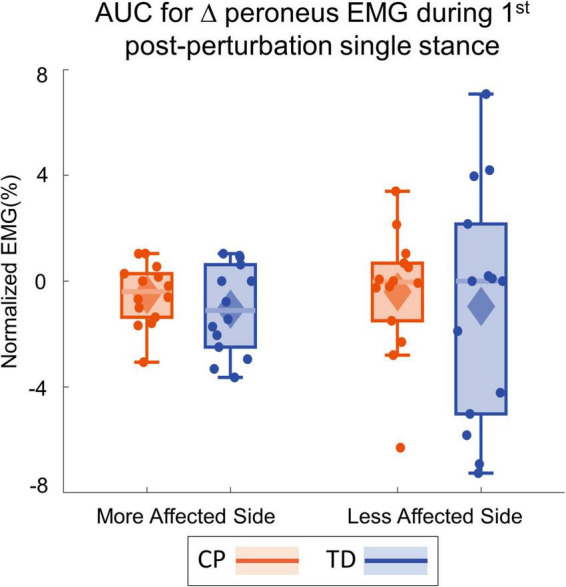
Box and whisker plots, with scattered dots indicating each subject, for AUC Δperoneus EMG for CP (orange) and TD (blue) groups on the more affected **(left)** and less affected **(right)** side.

Figure 4 shows the group average trajectories subtalar angle and Figure 5 shows the box and whisker plots for the AUC for subtalar angle for the stance foot during the first single stance following the visual perturbation. The integrated subtalar angle showed a significant group by side interaction (*p* = 0.047). *Post hoc* comparisons revealed that both CP and TD groups exhibited a similar increase in subtalar inversion on the less affected side (*p* = 0.221). On the more affected side, however, the TD group showed a significantly higher inversion at the subtalar joint compared to the CP group (*p* = 0.003). This response in the ankle roll mechanism is supported by the peroneal EMG data, which showed a localized reduction in peroneal activity during the first post-perturbation single stance in the more affected side of the TD group. Reduced peroneal EMG activity is indicative of reduced eversion and in turn suggestive of increased inversion. Figure 6 shows the group average trajectories for peroneal EMG and Figure 7 shows the box and whisker plots for the AUC of peroneal EMG for both groups. There was a greater reduction in peroneal muscle activity in TD compared to the CP group, however, this between group difference was not significant (*p* = 0.458).

### Foot placement response

**FIGURE 8:**
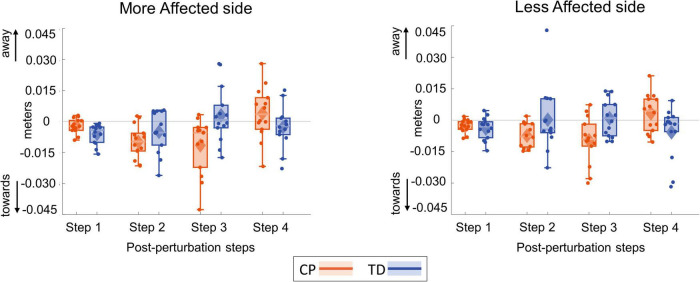
Box and whisker plots for foot placement response for the first four steps following a visual fall perturbation in CP (orange) and TD (blue). Thick gray line along zero at *X*-axis indicates the mean of control (no fall stimulus) steps, which is subtracted from perturbation data.

**FIGURE 9:**
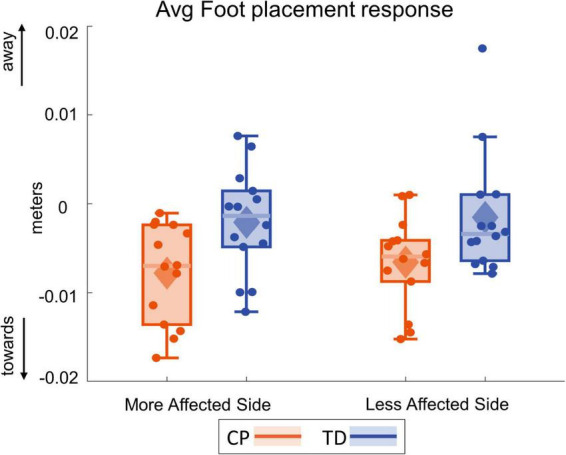
Box and whisker plots, with scattered dots indicating each subject, for average foot placement response over first three post-perturbation steps for CP (orange) and TD (blue) groups on the more affected **(left)** and less affected **(right)** side.

Figure 8 shows the foot placement changes for the first four steps following the visual perturbation. Figure 9 shows the box and whisker plots for the average foot placement response across the first three post-perturbation steps. At the first post-perturbation step, the TD group showed a greater foot placement response toward the direction of the fall stimulus compared to the CP group on both sides. This difference between the CP and TD groups was small (∼4 and 1 mm respectively on the more affected and less affected side, Figure 8) and was not statistically significant (*p* = 0.058). The average foot placement response over the first three post-perturbation steps, however, showed a significant group effect (*p* = 0.007). The CP group showed a higher foot placement response compared to the TD group over the first three post-perturbations steps, indicating a higher overall foot placement response in the CP group.

## Discussion

Our study investigated the role of visual input in walking balance control in individuals with CP and age- and sex-matched peers. We used virtual reality to induce visual fall stimuli and observed the resulting lateral shift in the whole-body CoM and the immediate responses in the foot placement and ankle roll balance mechanisms that generate the whole-body response. Our study provides compelling evidence that individuals with CP rely more on visual information compared to typically developing peers. As hypothesized, visual perturbations induced significantly larger CoM responses in the CP group compared to the TD group. Furthermore, the CP group used the foot placement balance mechanism more and the ankle roll balance mechanism less.

Participants moved their body away from the direction of the visual fall, as would be an appropriate response if they were actually falling during walking in a real-life environment. For example, if you bump into someone and are pushed to the right by the impact, then you would want to move your body to the left to arrest the fall. With the exception of the three participants from CP group that were excluded from the analysis, both CP and TD groups demonstrated this CoM shift in the away direction. In the CP group, this CoM response was larger and peaked later than in the TD group. This increased CoM shift is similar to the magnified sway response seen in standing balance studies in individuals with CP (Barela et al., 2011; Yu et al., 2020), and in older adults (Slaboda et al., 2011). Older adults showed a similarly magnified and variable CoM excursion response to continuous visual perturbations during walking (Franz et al., 2015). The magnified CoM response in the above studies has been attributed to problems in sensory re-weighting, related to age or pathology. Our results extend these hypotheses to walking balance control in individuals with CP. The magnified CoM response during walking implies that individuals with CP are more affected by changes in the visual environment and suggest increased reliance on vision over sensory modes for walking balance control.

In addition to differences in the overall CoM movement, we also investigated differences in the balance mechanisms used to generate the whole-body movement by modulating force against the ground. As hypothesized, individuals with CP demonstrated reduced ankle response and magnified foot placement response. The TD group showed ankle roll and foot placement responses that were similar to that of typical healthy adults (Reimann et al., 2018b). They showed an ankle roll response in the first step after the onset of the visual perturbation. In contrast, in individuals with CP the ankle roll response was reduced compared to TD. With respect to immediate foot placement response, the foot placement response in the CP group was also lesser than that of the TD group. However, the CP group did show a large foot placement response in the second and third post-stimulus steps. Thus, their overall average response over the first three steps was more magnified compared to the TD group. In summary, the TD group were able to return their response to their “normal” no perturbation gait after the first post-perturbation step. In contrast, the CP group had an overall increased foot placement response across the first three steps before they could return to their normal no-perturbation gait.

While we expected the participants with CP to perform worse on the affected side compared to the TD peers, we only found a group by side interaction for subtalar angle i.e., the CP group did significantly worse compared to the TD on affected side. We found a group effect for most of our variables, including the primary outcome measure of AUC COM excursion and Peak time as well as the secondary outcome measures of foot placement, i.e., irrespective of their side, the CP group overall did significantly worse compared to the TD group. A potential reason is that we had only had four individuals with hemiplegia in our study while the remaining participants had diplegia. A higher number of individuals with hemiplegia would have led to starker differences between the two sides.

Individuals with CP had reduced responses in the ankle roll mechanism and increased responses in the foot placement mechanism. Studies in standing balance have shown that individuals with TD use a distal to proximal organization of balance responses to mechanical perturbations, with the ankle being recruited first and the hip last. Individuals with CP tend to use a different pattern that favors the proximal joints, producing multiple bursts of torque at hip, knee and ankle joint simultaneously, indicating that they are unable to make quick, precise and finer adjustments through distal control at the ankle (Chen and Woollacott, 2007). Our results provide evidence of similar preference of proximal over distal strategies during walking balance control in CP.

Another potential reason for the reduced ankle roll is the higher cadence and reduced step time in the CP group compared to the TD group. In neurotypical adults, the dominant balance response shifts between the ankle roll and foot placement mechanism depending on the walking cadence (Fettrow et al., 2019a). Lower cadences lead to longer single stance duration, with longer time to apply force using the ankle roll mechanism, which reduces the need for a foot placement response. Conversely, walking at higher cadences results in lower single stance duration, leading to reduced opportunity for the ankle roll mechanism. At the same time, frequent foot placements at higher cadences provide more opportunity for foot placement control. Our cohort of individuals with CP walked with similar velocity, but at a higher average cadence. It is possible that the differences in balance mechanisms between groups are a consequence of this difference in cadence, i.e., that people with CP use less ankle roll because they walk at higher cadence.

It is also possible that the magnified CoM response in CP is due to motor deficits. Motor impairments at the ankle, such as inability to generate appropriate and timely muscle contraction due to spasticity, could lead to the observed lack of ankle roll use in the CP group. While we excluded individuals with severe spasticity (Modified Ashworth Scale > 4), mild spasticity might still impair the relatively precise modulations required for the ankle roll mechanism. Our hypothesis that impaired somatosensory and proprioceptive processing causes balance problems in CP predicts the increased CoM response and altered balance mechanisms we observed here, but other explanations, such as motor deficits or increased cadence in CP precluding use of the ankle roll mechanism cannot be excluded by our results. Interestingly, if it is true that individuals with CP do not have access to the ankle roll due to motor impairments, this might *cause them* to walk with higher cadence, because stability at lower cadence depends on the ankle roll mechanism, which they do not have access to. More research is needed to distinguish between these possible explanations of the observations in this study.

Lastly, visual impairments in individuals with CP can broadly be due to ocular disorders or due to a non-ocular, brain-based processing disorder, i.e., CVI (Ego et al., 2015; Philip et al., 2020; VerMaas et al., 2020). While we excluded any individuals with any ocular impairments as well as known diagnosis of CVI, given how complex and challenging the diagnosis of CVI is, it is possible that some individuals in the study had impaired processing of visual information. Previous research has shown that children with CP were slower to detect a directional change in visual motion and made more errors in identifying the change compared to the control group (VerMaas et al., 2019). These higher reaction times in individuals with CP could be a potential reason behind the CP group in our study having higher peak times compared to their TD peers. However, despite potential visual impairments, these individuals had more magnified responses to visual perturbations, as evidenced by higher AUC COM excursion, and thus, relied more on vision for balance control. This may indicate that despite impaired processing of both visual and proprioceptive information, individuals with CP prioritized visual input over proprioceptive input for walking balance. While proprioceptive deficits at the lower extremity have been related to standing balance (Zarkou et al., 2020), it is likely that a similar relationship may exist between visual reliance and proprioceptive deficits. Thus, those individuals with greater proprioceptive deficits may show a greater reliance on vision. In such individuals, assistive walking devices would not only serve its primary biomechanical function of improving base of support, reduction of lower limb loads and providing propulsion and braking but it may help in augmentation of somatosensory cues by providing additional information about the spatial orientation of the body (Bateni and Maki, 2005).

## Limitations

Our study only included individuals with CP who were able to independently ambulate without any walking aids for multiple bouts of 2 min (GMFCS levels I and II), mainly to reduce the possibly confounding influence of tactile or visual feedback from handrails during visually perturbed walking. Individuals with lower functional mobility may respond differently to visual perturbations while walking. Second, there may be some baseline differences in the walking characteristics of individuals with CP and TD that are secondary to the neuromotor deficits due to CP. E.g., individuals with CP walked with a higher cadence and shorter step time compared to those with TD. It is possible that such differences, in addition to neuromotor deficits, contribute indirectly to the group differences in responses to visual perturbations observed here. Probing whether the group differences are due to neuromotor deficits only or due to the differences in gait characteristics of CP and TD group, however, is beyond the scope of this study and should be subject of future research.

## Conclusion

Individuals with CP demonstrated a magnified and delayed response to visual perturbations. They were more affected by changes in visual cues and relied more on visual information for walking balance control. Our findings suggest that individuals with CP may alter the relative contribution of visual input in active control of walking balance, commonly referred to as sensory reweighting (Peterka, 2002). Also, individuals with CP showed increased use of the foot placement mechanism for balance control and diminished use of the ankle roll mechanism. This suggests a reliance on proximal over distal control of walking balance in individuals with CP, similar to what other studies observed in standing. These findings provide insight into how sensory information is processed for balance control during walking by individuals with CP and which motor responses they prefer to adopt in response to perceived threats to their balance. This information will be critical in planning treatment targeted toward addressing specific deficits in walking balance and fall prevention in CP. The current standard of care for CP largely focuses on motor-related deficits such as muscle weakness, spasticity, contractures, and reduced flexibility with mixed outcomes (Pin et al., 2006; Blumetti et al., 2012; Dreher et al., 2012; Taylor et al., 2013). Our findings highlight the need for sensory-based therapeutic approaches that address somatosensation and proprioception in addition to the current motor-centric treatments to provide a more comprehensive approach to balance rehabilitation in individuals with CP.

## Data availability statement

The datasets generated and analyzed in this study can be found at: https://doi.org/10.5061/dryad.1ns1rn8x2.

## Ethics statement

The studies involving human participants were reviewed and approved by the University of Delaware Institutional Review Board. Written informed consent to participate in this study was provided by the participants or their legal guardian/next of kin.

## Author contributions

AS, HR, and SL: conception and design of the work. AS, MA, and HR: data collection and critical revision. AS and HR: analysis of data and interpretation. AS: drafting the work. AS, MA, SL, JJ, and HR: revision and final approval of the work. All authors agreed to be accountable for the content of the work.
