# Supplementary figures and images for: Individuals with cerebral palsy show altered responses to visual perturbations during walking
Source: Front Hum Neurosci. 2022 Sep 8;16:977032. doi: 10.3389/fnhum.2022.977032 (PMC9493200; doi:10.3389/fnhum.2022.977032)

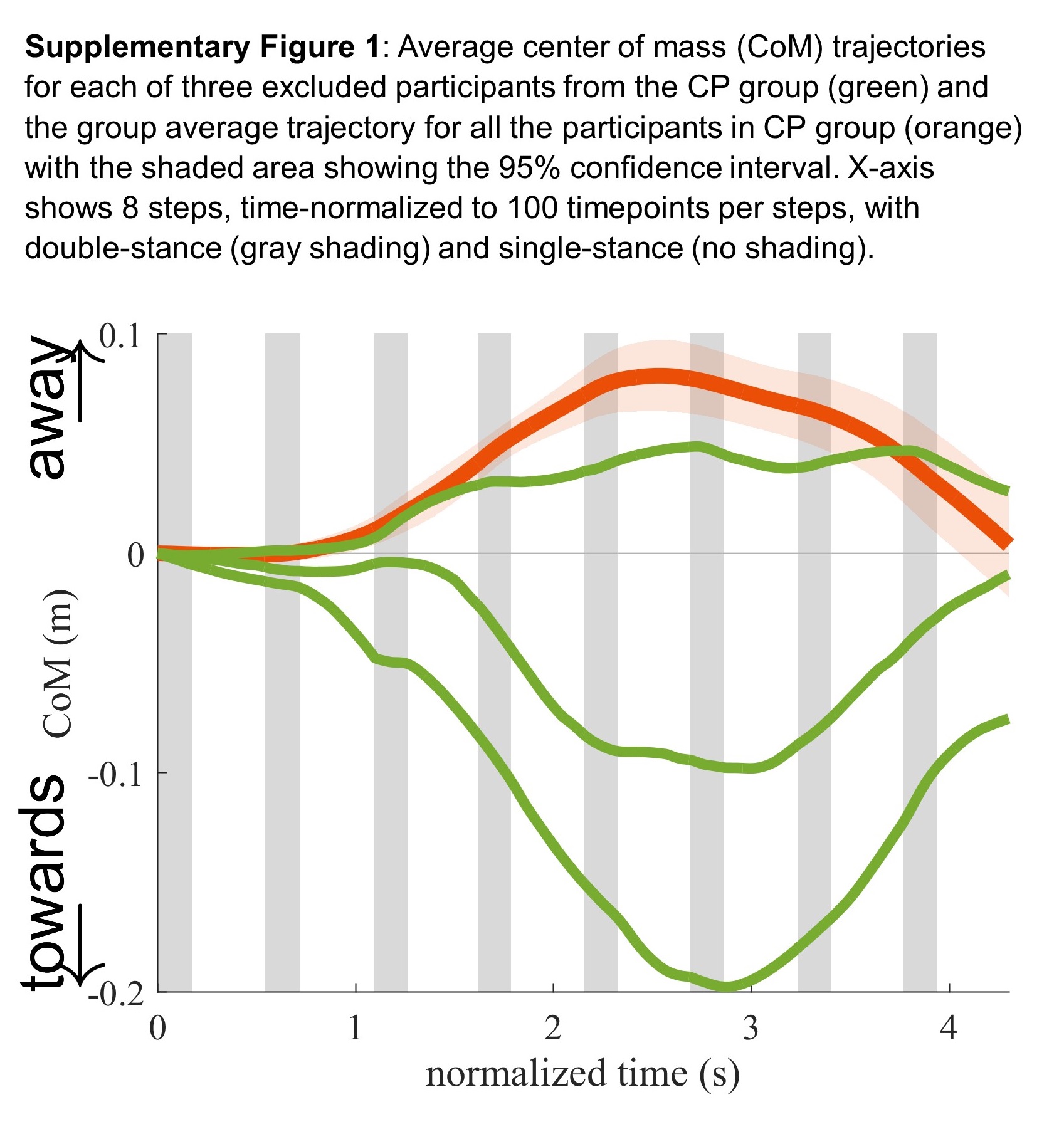

Supplement: Supplementary file 1 [file Image_1.jpg]
