# Supplementary material for: Individuals with cerebral palsy show altered responses to visual perturbations during walking
Source: Front Hum Neurosci. 2022 Sep 8;16:977032. doi: 10.3389/fnhum.2022.977032 (PMC9493200; doi:10.3389/fnhum.2022.977032)

Table S1: Statistical analysis for within-subject (side and group*side) and between-subject (group) effects

| Outcome Measure | Effect | df | F statistic | p value | Partial eta squared |
| --- | --- | --- | --- | --- | --- |
| AUC M-L COM excursion (meters*sec) | group | [1,26] | 6.451 | **0.017** | 0.199 |
|  | side | [1,26] | 2.055 | 0.164 | 0.073 |
|  | group*side | [1,26] | 2.284 | 0.143 | 0.081 |
| Peak M-L COM excursion (meters) | group | [1,26] | 3.037 | 0.093 | 0.105 |
|  | side | [1,26] | 1.582 | 0.220 | 0.057 |
|  | group*side | [1,26] | 2.636 | 0.117 | 0.092 |
| Peak Time (seconds) | group | [1,26] | 5.193 | **0.031** | 0.166 |
|  | side | [1,26] | 4.585 | **0.042** | 0.150 |
|  | group*side | [1,26] | 0.725 | 0.402 | 0.027 |
| Foot Placement-1st step (meters) | group | [1,26] | 3.921 | 0.058 | 0.131 |
|  | side | [1,26] | 1.342 | 0.257 | 0.049 |
|  | group*side | [1,26] | 3.729 | 0.064 | 0.125 |
| Foot Placement-averaged over 3steps (meters) | group | [1,26] | 8.604 | **0.007** | 0.249 |
|  | side | [1,26] | 0.385 | 0.540 | 0.015 |
|  | group*side | [1,26] | 0.080 | 0.780 | 0.003 |
| Subtalar angle (degrees) | group | [1,26] | 4.017 | 0.056 | 0.134 |
|  | side | [1,26] | 0.056 | 0.814 | 0.002 |
|  | group*side | [1,26] | 4.336 | **0.047** | 0.143 |
| Peroneal EMG (%) | group | [1,23] | 0.571 | 0.458 | 0.024 |
|  | side | [1,23] | 0.030 | 0.865 | 0.001 |
|  | group*side | [1,23] | 0.000 | 1.000 | 0.000 |

Figure S1: Center of Mass excursion (CoM) trajectories for the three individuals with CP that were excluded from the study (green) and the average CoM excursion of the 14 participants in the CP group (orange). Shaded areas depict 95% confidence interval for the CP group.


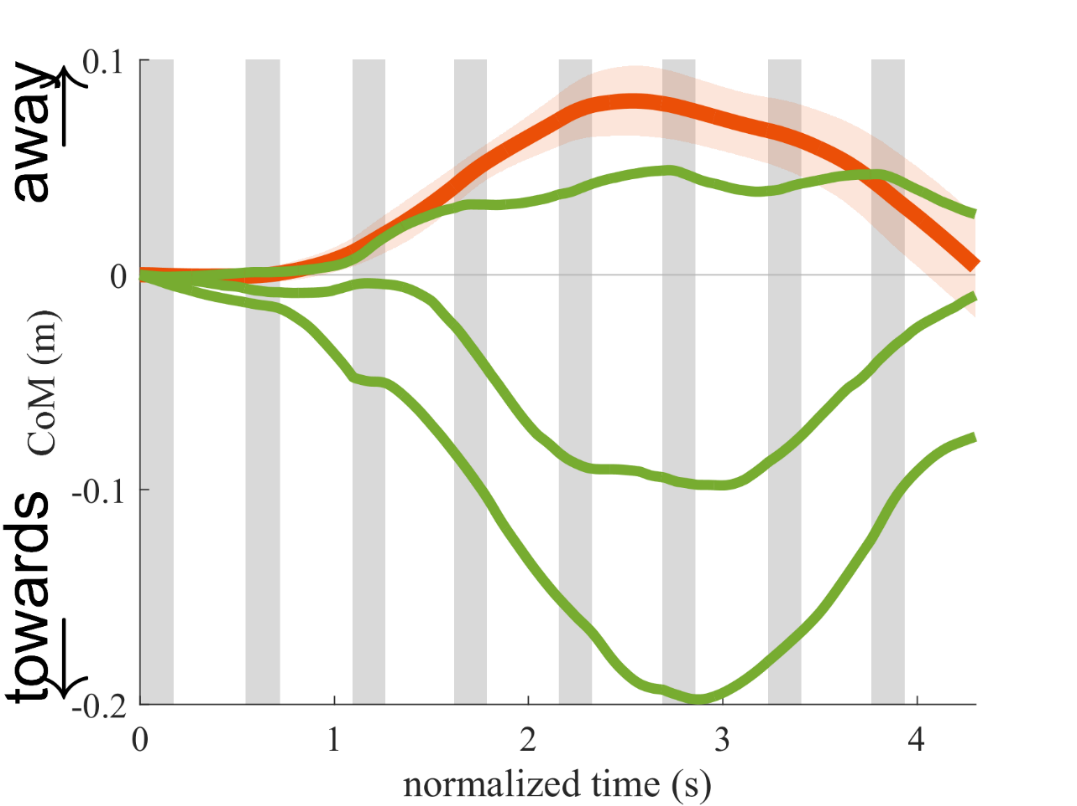

Supplement: Supplementary file 2 [file Data_Sheet_1.docx]
